# Genome-wide analyses of Mycobacterium tuberculosis complex isolates reveal insights into circulating lineages and drug resistance mutations in The Gambia

**DOI:** 10.21203/rs.3.rs-5913893/v1

**Published:** 2025-02-27

**Authors:** Leopold Tientcheu, Fatou Faal, Naffie Top, Olimatou Jobe, Sang Marie Colley, Abigail Ayorinde, Alieu Mendy, Binta Sarr-Kuyateh, Simon Donkor, Martin Antonio, Bouke de Jong, Andrea Rachow, Beate Kampmann, Jayne S. Sutherland, Hongwei Li, Tom Blundell, Susana Campino, Thomas Kohl, Viola Dreyer, Stefan Niemann, Arun Pandurangan, Taane Clark, Jody Phelan

**Affiliations:** MRC Unit The Gambia at the London School of Hygiene & Tropical Medicine; Medical Research Council The Gambia Unit; Institute of Tropical Medicine; LMU Munich; The Gambia at the London School of Hygiene and Tropical Medicine, Vaccines & Immunity Theme; London School of Hygiene and Tropical Medicine; Research Center Borstel - Leibniz-Center for Medicine and Biosciences; Research Center Borstel - Leibniz-Center for Medicine and Biosciences; London School of Hygiene & Tropical Medicine; London School of Hygiene and Tropical Medicine

## Abstract

Tuberculosis (TB), caused by the *Mycobacterium tuberculosis* complex (MTBC), remains a pressing global health challenge, with the West African region, including The Gambia, experiencing a substantial burden. This study explores the genetic diversity of MTBC strains circulating in The Gambia for nearly two decades (2002–2021) to enhance understanding of drug resistance dynamics and inform targeted diagnostic and treatment strategies. Using whole-genome sequencing (WGS) data from 1,803 TB isolates, we identified the predominance of lineage 4 (L4, 67.2%) and lineage 6 (L6, 26.6%) strains, with L4 showing more significant genetic variability over time. Drug susceptibility analysis of these isolates revealed that 78% (1421 isolates) were drug-susceptible, while 6.5% (119 isolates) exhibited resistance, primarily to isoniazid, rifampicin, and their combination. Additionally, 15.5% (282 isolates) were classified as Other, having potential drug-resistance mutations of uncertain significance by the WHO catalogue. Interestingly, our resistance-associated analysis showed the lineage 6 specific ethambutol uncertain significance (by WHO catalogue) mutation (embC Ala307Thr) more prevalent in The Gambia than in West Africa and globally. Structural analysis showed that first-line drug resistance mutations frequently occur in solvent-inaccessible and conserved regions of proteins, often impacting protein stability and reflecting a balance between resistance, fitness, and evolutionary adaptation.

This study highlights the coexistence of globally prevalent and regionally restricted MTBC lineages, underscoring the importance of region-specific TB control measures. Integrating bioinformatic and structural analyses revealed many uncertain significant mutations by the WHO catalogue in The Gambian isolates compared to West Africa and globally. These findings reinforce the necessity of continuous genomic surveillance to address the evolving challenges of TB in high-burden settings like West Africa.

## Introduction

Tuberculosis (TB) caused by bacilli of the *Mycobacterium tuberculosis* complex (MTBC) remains a significant public health problem worldwide. In 2022, an estimated 10.6 million people developed TB, with 1.3 million deaths reported. West Africa accounted for 10% of global TB deaths in 2022, including 3,900 cases and 580 fatalities in The Gambia. TB disproportionately affects resource-limited communities and low-income countries ^1^.

Whole genome sequencing (WGS) advances have greatly enhanced our understanding of MTBC lineage diversity and phylogeographical distribution ^2, 3^. Of the ten known MTBC lineages (L1-L10) (Guyeux et al., 2024), all are found in Africa. In West Africa, TB is driven by *M. tuberculosis* sensu stricto *(Mtb)* and *M. africanum (Maf)* lineages, which co-exist in affected populations ^4^. While geographically restricted lineages like *M. africanum* (L5, L6) are endemic to West Africa, globally disseminated lineages such as Mb-Beijing (L2) and Mb-Europe-America (L4) are also prevalent (Galagan et al., 2014).

West Africa follows the World Health Organization (WHO) guidelines for TB treatment, involving a prolonged multi-drug regimen ^1^. TB patients’ treatment encompasses two regimens: drug-susceptible (DS) and -resistant (DR). DS-TB patients undergo a six-month treatment course comprising an intensive phase with rifampicin (RIF), isoniazid (INH), ethambutol (EMB), and pyrazinamide (PZA) for two months, followed by a four-month continuation phase with RIF and INH ^5^. For DR-TB cases, treatment regimens are adjusted based on resistance profiles, often requiring second-line drugs and extended treatment durations, which complicate patient management and result in poorer treatment outcomes (Seaworth, 2017).

Previous studies have shown that TB patient responses to standard anti-TB treatment vary depending on the infecting MTBC lineages, notably their immune response ^6, 7, 8, 9, 10^. Moreover, hypervirulent strains can dampen immune defences, leading to accelerated disease progression and increased transmission rates ^11^.

The mutation rates of lineages have been found to vary significantly, with some lineages exhibiting a higher propensity for developing mutations that confer resistance to primary TB drugs ^12^. For example, L2 has been noted for its increased mutation rates, particularly in drug-resistant (DR) strains, contributing to intrinsic treatment challenges and the emergence of multidrug-resistant (MDR) TB ^13, 14, 15^ The persistence and proliferation of resistant strains during treatment can lead to therapeutic failures and complicate TB control efforts ^16^.

This study investigates the genetic diversity of MTBC strains circulating in The Gambia over nearly two decades (2002 to 2021) and explores their implications for effective TB management. By integrating structural bioinformatics and computational approaches, we analysed the most extensive WGS collection of MTBC isolates in a West African country. We present the abundance, distribution and effects of genetic mutations on drug-target protein stability and conservation properties. Understanding these genetic variations is crucial for designing new drugs and developing effective TB treatment strategies, particularly in regions like West Africa, where multiple MTBC lineages coexist.

## Methodology

### Ethical statement

This study received ethical approval from the Medical Research Council Unit, The Gambia, at the London School of Hygiene and Tropical Medicine (MRCG@LSHTM)/The Gambian Government joint ethical committee. All recruited study participants or guardians provided written informed consent.

### Sequencing and epidemiological data

The whole genome sequences (WGS) and epidemiological data used in this analysis (n=1803) were sourced from consecutive TB projects hosted by the TB case contact platform at the MRCG@LSHTM between 2002 and 2020. These projects include the following with their respective number of WGS samples: PRJEB53138 (Enhance Case Finding; n=1302), SCC 1289 (Childhood TB Program; n=234), SCC 1523 (TB Sequel; n=216) and Recurrent TB (n=52). The isolate’s metadata used in this study included age, sex and collection year.

### Microbiology and DNA Extraction

Briefly, stored MTBC isolates from archived stocks or directly from a microbiology growth indicator (MGIT^™^) positive tube were subcultured into Middlebrook 7H9 broth or Lowenstein-Jensen (LJ) slopes to multiply the colonies. Genomic DNA was extracted using the cetyltrimethylammonium bromide (CTAB) method, as previously described ^17^. The extracted DNA underwent WGS on the Illumina HiSeqX platform at the Forschungszentrum Research Center Borstel, Germany. For the PRJEB53138 isolates, the DNA was extracted using Maxwell^®^ 16 Viral Total Nucleic Acid Purification Kit (Promega Corporation, Fitchburg, WI, USA) following the manufacturer’s instructions and sequenced in MicrobsNG in the United Kingdom.

### Bioinformatic and phylogenetic analysis

Raw sequence data (approximately 2000 samples) was processed and analysed to ensure data quality and accuracy. The Kraken2 database tool was used to filter contaminated sequences and exclude non-MTBC strains. Poor-quality reads were trimmed using Trimmomatic (v0.39) with the following parameters: LEADING:3 TRAILING:3 SLIDINGWINDOW:4:20 MINLEN:36. The quality of the processed reads were reassessed using FastQC.

For each sample, trimmed reads were aligned to the *Mycobacterium tuberculosis* H37Rv reference genome (accession: NC_000962.3) using BWA-MEM software ^18^. Single nucleotide polymorphisms (SNPs) and insertions/deletions (indels) were identified through the application of the Genome Analysis Toolkit (GATK) ^19^ and Sequence Alignment Map (SAM) ^20^ tools. Genomic VCF files from all the samples were merged, and multi-FASTA alignments were generated using BEDTools software.

Phylogenetic relationships between the samples were inferred by constructing a phylogenetic tree with IQ-TREE software ^21^. The tree was visualised and annotated using the Interactive Tree of Life (iTOL) v6 software platform ^22^. The tree was visualised and annotated using the iTOL software platform. MTBC lineages and genotypic drug resistance profiles for each isolate were determined using the TB-Profiler pipeline (v4.4.0; database version: e25540b) ^23^. Variants, including missense and frameshift mutations within known drug resistance loci, were analysed and compared to established databases such as TB-Profiler and the WHO catalogue to identify reported and potential unreported polymorphisms. Mutations in Tier1 genes for The Gambian dataset were compared with global mutation data (>100K mutations) and specific datasets from other West African countries ^24, 25^.

The analysis included detailed information for each mutation, including the gene names, nucleotide changes, mutation frequency, and count for each country, identified by their country codes. This comprehensive approach provided insight into regional and global genetic diversity and drug resistance dynamics in MTBC strains.

### Protein structural modelling and mutant stability prediction

Missense mutations associated with first-line drugs (RIF, INH, PZA, and EMB) were filtered using a frequency cutoff of 80% to ensure accuracy and relevance. Redundant mutations within the same gene were identified and removed to clarify the dataset and eliminate duplication. The final dataset comprised 943 isolates and their associated missense mutations, of which 614 were classified as susceptible and 329 as resistant. This curated dataset offers a comprehensive overview of the genetic basis of drug susceptibility and resistance.

Protein structures for the target genes were obtained from the Protein Data Bank (PDB), a key repository of experimentally determined macromolecular structures critical for biomedical research and drug discovery ^26, 27^. This dataset included four PDB files derived from experimentally determined crystal structures and twenty predicted structures using AlphaFold, a protein structure prediction tool ^28^. To predict the stability changes caused by mutations, the Delta Delta G (ΔΔG), representing the difference in free energy between wild-type and mutant protein forms, was calculated using PyRosetta, FoldX, and site-directed mutator (SDM) (https://compbio.medschl.cam.ac.uk/sdm2/) ^29, 30, 31^.

ConSurf (https://consurf.tau.ac.il/consurf_index.php) was employed to evaluate the evolutionary conservation of amino acids at mutation sites. Conservation grades, ranging from 1 (highly variable) to 9 (highly conserved), were assigned based on the evolutionary significance of specific residues ^32^. Highly conserved residues are often critical for structural integrity or functional roles in proteins. The conservation grades for mutation positions were extracted and compared between mutations classified as resistant and susceptible. This comparison offered insights into the mutations’ evolutionary importance and potential functional consequences.

## Results

### Study Demography

This study analysed 1803 MTBC isolates with whole-genome sequencing (WGS) data from TB patients residing in the Greater Banjul Area, which accounts for 80% of all TB cases in The Gambia. Metadata, including either age, sex, or year of sample collection, was available for 1713 isolates (95%) (Table 1). The majority of isolates (1145/1585, 72.2%) were from male patients, with the highest representation from individuals aged 18–29 (498, 32%) and 30–44 (409, 26.3%) years. Most samples were from patients diagnosed between **2012 and 2015** (1433/1713, **83.6%**) (**S1 figure**).

### MTBC lineages

**Figure 1:**
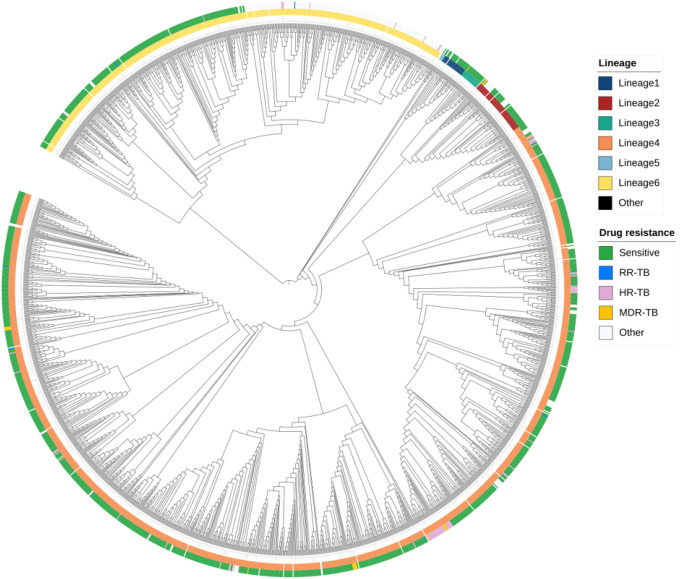
Phylogenetic relationship and drug resistance profile of 1803 MTBC isolates. The outer circle represents drug susceptibility output based on genotyping: drug-sensitive isolates (Green), Rifampicin mono-resistant (RR-TB; blue), Isoniazid mono-resistant (HR-TB; Pink), multi-drug-resistant (MDR; Yellow), and isolates resistant to other drugs (Other; White). The inner circle consists of MTBC lineages: Lineage 1 (Dark blue), Lineage 2 (Brown), Lineage 3 (Green), Lineage 4 (Orange), Lineage 5 (Light blue), Lineage 6 (Yellow) and Other (Black). The branches represent a clustering of isolates based on the SNP’s differences between them.

Phylogenetic analysis revealed the clustering of isolates by lineage, confirming the dominance of specific MTBC lineages in the population (Figure 1). Most isolates (94%) belonged to *Mb* Lineage 4 (L4; 1214/1804, 67.2%) and *Maf* Lineage 6 (L6; 480/1804, 26.6%) (Table 1). L4 has remained the predominant lineage throughout the study period (**S1 figure**). Among the sub-lineages, L4.1 (410 isolates) and L4.3 (LAM; 323 isolates) were the most abundant within L4, while L6.1 was the most common sub-lineage of L6 (**S2 figure**).

### Drug resistance

The genotypic resistance profile revealed that 1421 (78%) were drug-susceptible (DS). Among the drug-resistant (DR) isolates, 90 (5.0%) were resistant to INH alone, 10 (0.6%) were resistant to RIF alone, and 19 (1.1%) were multi-drug resistant (MDR). Additionally, 282 isolates (15.3%) were classified as other, having potential drug-resistance mutations, identified by the TB-Profiler pipeline ^23^. The most frequent mutation underlying resistance to INH was *katG*Ser315Thr, observed in 71 isolates (71/90, 78.8%) (Table 2). Lineage-specific mutations were also detected in known drug-resistance genes. For instance, *embC* Ala307Thr, a mutation associated with ethambutol but classified as uncertain significant by the WHO catalogue, was specific to L6, with a frequency of 56.34% (270/480). Another ethambutol-associated uncertain significant mutation, *embA* Thr113Arg, was also unique to L6 with a frequency of 22.61% (106/480) (Table 2). According to the drug resistance classification, INH resistance (HR-TB) and MDR-TB were predominantly observed in lineage 4. In contrast, lineage 6 exhibited the highest number of resistances classified as “Other,” including mutations of uncertain significance defined by the WHO catalogue. INH and these “Other” resistances were mainly detected between 2012 and 2019 (**S3 figure**).

### Genetic variability

**Figure 2:**
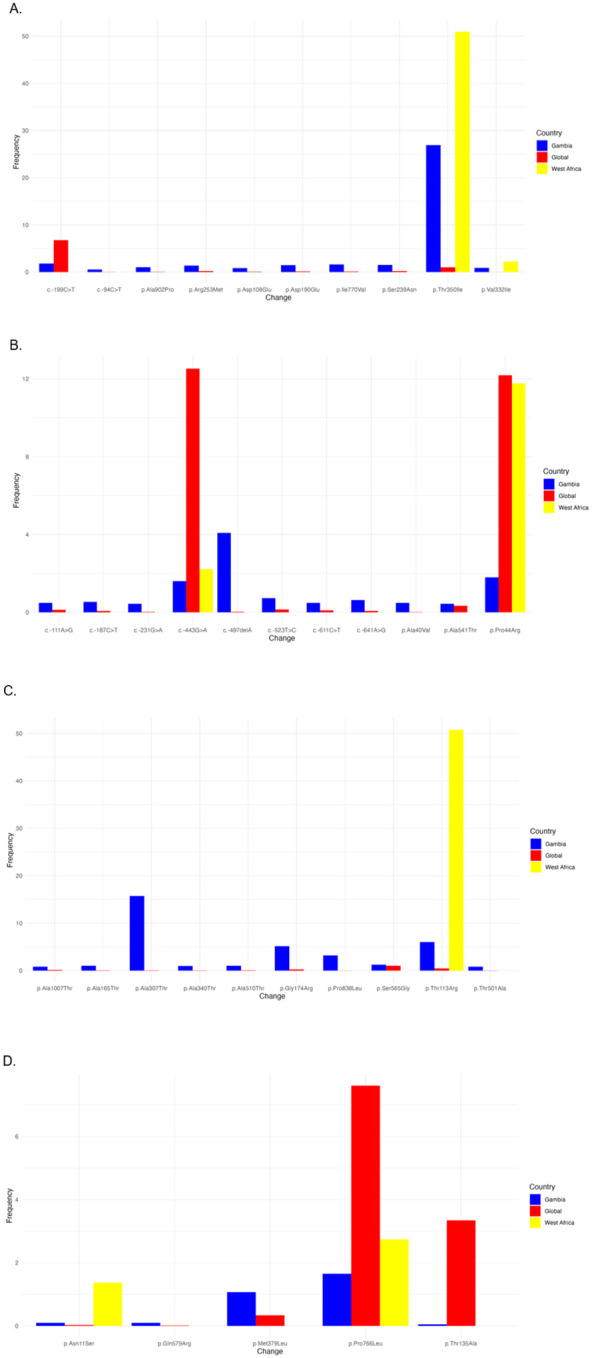
Diversity and prevalence of the WHO catalogue’s mutations, with uncertain significant mutations in the first-line drug-targeted genes across The Gambia, West Africa, and the global dataset. In each figure, the Y-axis shows the frequency of each mutation displayed on the X-axis in the three regions of interest: The Gambia (Blue), West Africa (Yellow), and the rest of the globe (Red) for the first-line drugs, including (A) Rifampicin, (B) Isoniazid, (C) Ethambutol, and (D) Pyrazinamide.

To investigate the specificity of genetic mutations in MTBC isolates from The Gambia, we compared missense mutation frequencies in Gambian isolates to those in the rest of West Africa and global datasets. Among over 100,000 mutations identified across 100 countries, 12,411 mutations in all drug resistance genes (11.6%) originated from The Gambia, underscoring the country’s notable contribution to the global mutation pool. Mutations in genes associated with drug resistance were found at significantly higher frequencies in The Gambia compared to global averages, particularly in key genes such as rpoB (RIF resistance), inhA (INH resistance), and embB (EMB resistance). For example, the rpoB Thr350Ile uncertain significant mutation by the WHO catalogue was observed in 26.9% of Gambian isolates compared to 0.99% globally (Figure 2A). The embC Ala307Thr uncertain significant mutation appeared in 15.7% of Gambian isolates but was rare globally (Figure 2C). These mutations were steadily identified in MTBC isolates over the study period (**S4 figure**). In contrast, uncertain significant mutations associated with resistance to second-line drugs, such as moxifloxacin, were more common in The Gambia and West Africa than the global average. For example, gyrB Ala403Sep and gyrA Leu398Phe were both found at higher frequencies in The Gambia (34% and 29%) and West Africa (41% and 31%), respectively, compared to the global frequency (**S5 figure**). Some non-resistance mutations, like c.−100C>T, were uniquely prominent in The Gambia, often co-occurring with ethambutol-resistance (**S6 figure**).

### Structural and conservation properties of resistance and susceptible mutations

**Figure 3:**
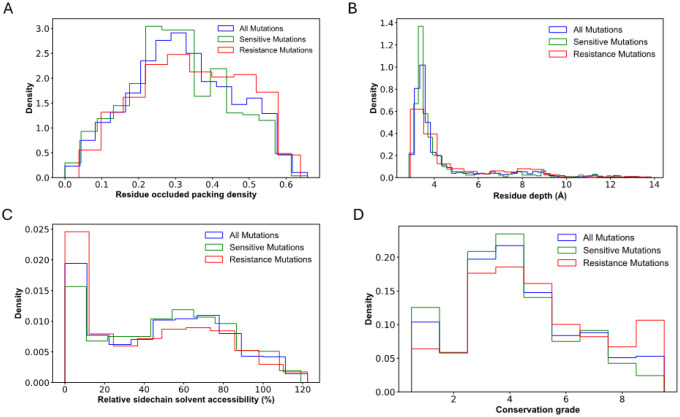
Structural and conservation properties for resistant, susceptible and background mutations. (A) Residue occluded packing density, (B) Residue depth, (C) Relative side chain solvent accessibility and (D) Residue conservation. The line pattern shows the density of each mutation type for the different parameters evaluated.

The distributions and structural properties of mutant sites were analysed based on residue depth, occluded surface packing (OSP), and relative solvent accessibility (RSA). A significant difference was observed between resistance and susceptible mutations in all categories of the structural properties (two-tailed Mann-Whitney test, p<0.05). It is interesting to note that the resistance and susceptible mutations occur more frequently in tightly (OSP > 0.4) and less tightly packed (OSP < 0.4) environments in the protein structure, respectively (Figure 3A). In terms of residue depth, susceptible mutations were observed more often at shallow residue depths (< 4 Å), while resistant mutations were concentrated at greater depths (8–9 Å) (Figure 3B). Similar trends were also observed for RSA, where the resistance mutations were found at higher frequency in solvent-inaccessible regions (RSA < 20%). In contrast, susceptible mutations were more frequently located in solvent-accessible regions (Figure 3C).

Additionally, the conservation levels of resistance and susceptible mutations were analysed. Resistance mutations exhibited a significantly higher conservation level than background and susceptible mutations. Specifically, at the conservation grade from 5 to 9, the density of resistant mutations surpassed that of background and susceptible mutations. Conversely, at conservation grades of 1 to 4, the density of susceptible mutations was more prevalent than background and resistant mutations (Figure 3D). Overall, resistant mutations displayed more significant conservation than susceptible mutations (one-tailed Wilcoxon matched-pair signed rank test, p<0.05), highlighting their potential functional and evolutionary significance.

### Mutant stability prediction

**Figure 4:**
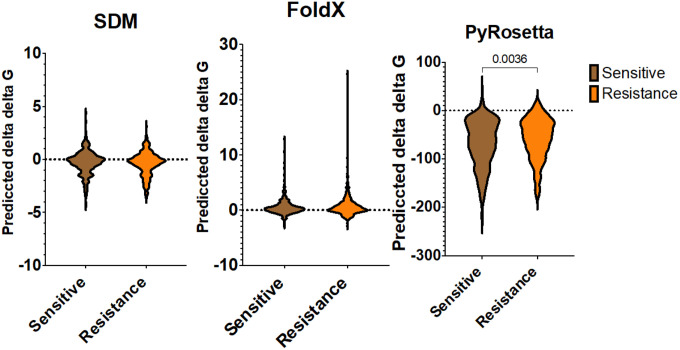
Predicted ΔΔG of overall resistant and susceptible mutations in first-line drugs. Comparison of the distributions of ΔΔG for SDM (A), FoldX (B) and PyRosetta (C) between resistant and susceptible mutations.

The values of the ΔΔG (change in free energy) were analysed to predict the impact of resistant and susceptible mutations on protein stability. No significant differences in ΔΔG were observed between resistance and susceptible mutation using FoldX and SDM (Figure 4). SDM predicted median ΔΔG values of −0.21 for resistance and −0.18 for susceptible mutations. On the other hand, FoldX predicted median ΔΔG values of 0.31 for resistance and 0.24 for susceptible mutations. Both tools suggested that these mutations could severely destabilise or stabilise the protein (absolute ΔΔG > 0.5).

In contrast, PyRosetta analysis revealed significant differences in ΔΔG values (two-tailed Mann-Whitney test, p<0.05). Resistant mutations showed a relatively more significant destabilising effect (median ΔΔG=−52.74) compared to susceptible mutations (median ΔΔG=−62.28) (Figure 4).

**Figure 5:**
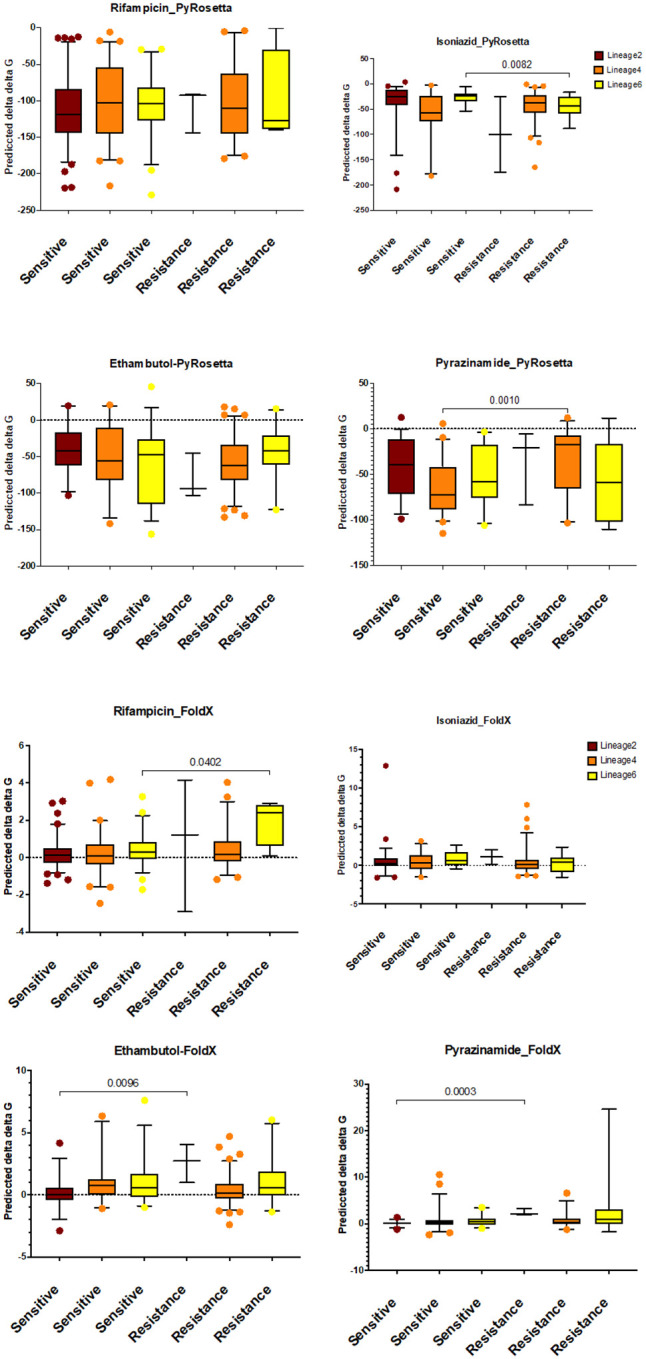
Predicted ΔΔG of resistant and susceptible mutations by first-line drug and MTBC lineages. Comparison of the distributions for (A) PyRosetta and (B) FoldX between resistant and susceptible mutations in lineage 2 (Brown), L4 (Orange) and L6 (Yellow).

To further investigate ΔΔG differences between resistant and susceptible mutations, mutations were grouped by first-line anti-tuberculosis drugs (RIF, INH, EMB, and PZA) and categorised by MTBC lineages. No significant differences between resistant and susceptible mutations were observed for any of the four drugs using SDM (**S7 figure**). In contrast, PyRosetta analysis revealed statistically significant differences (two-tailed Mann-Whitney test) for INH in Lineage 6 and EMB in Lineage 4 (Figure 5A). FoldX analysis also identified substantial differences in ΔΔG values for RIF in Lineage 6 and EMB and PZA in Lineage 2 (Figure 5B).

## Discussion

This study provides insights into the genomic landscape of *Mycobacterium tuberculosis* complex (MTBC) isolates in The Gambia for nearly two decades, shedding light on the country’s epidemiology, lineage diversity and drug resistance (DR) patterns. Consistent with prior studies in West Africa (De Jong et al., 2010), MTBC Lineage 4 (L4) and 6 (L6) were found to dominate, collectively accounting for 94% of TB cases.

L4, the most prevalent lineage globally, represented 67.2% of isolates, likely due to its high virulence ^33^, rapid progression to active TB ^34^, and adaptability to diverse environments ^35^. Within L4, the overrepresentation of sub-lineages L4.3 (LAM) and L4.1 further supports the role of these genetic clusters in driving its success in this population. LAM is a well-known lineage associated with increased virulence and transmission potential, which may explain its widespread distribution in this region ^36^

In contrast, L6, which accounted for 26.6% of the isolates, remains mainly geographically restricted to West Africa. This highlights the importance of region-specific interventions considering the local strains’ genetic makeup ^37^. Despite its limited global prevalence, this study’s significant presence of L6 aligns with its known confinement to the region. The predominance of sub-lineage L6.1 highlights its evolutionary adaptation and persistence within this population, likely shaped by unique host-pathogen interactions in the region ^38^.

The demographic trends observed are consistent with global TB patterns. Most cases were identified in male patients (72.5%), reflecting the higher incidence of TB among men worldwide ^1^. The patient’s age distribution, with the highest burden among individuals aged 18–29 and 30–44, underscores the significant impact of TB on economically and socially critical population segments in The Gambia. These findings emphasise the importance of targeted interventions to mitigate the disease’s socioeconomic impact in The Gambia.

The detection of 19 multidrug-resistant (MDR) isolates highlights the pressing challenge of TB in The Gambia, given the limited treatment options and increased risk of treatment failure associated with mistreated MDR TB. The higher prevalence of isoniazid (INH) mono-resistance (90isolates) comparedto rifampicin (RIF) mono-resistance (10 isolates) suggests that INH resistance may be an emerging issue in The Gambia. This finding underscores the need for continuous surveillance and updated treatment protocols that reflect the evolving resistance patterns. Furthermore, the study’s reliance on genotypic data to infer DR highlights the importance of integrating molecular diagnostics into routine TB control programs, which can facilitate the timely and accurate detection of DR strains. The high frequency of specific mutations, such as *rpoB* Thr350Ile and embC Ala307Thr (EMB), highlights the importance of region-specific genomic surveillance to guide TB control efforts effectively. Identifying mutations with uncertain significance in the WHO catalogue, particularly those with higher frequencies in The Gambia, highlights a critical knowledge gap. Investigating these mutations’ clinical relevance, fitness effects, and contribution to DR through in vitro and in vivo studies will enhance our understanding of DR mechanisms.

Analysis of structural properties revealed key differences between resistant and susceptible mutations. Resistant mutations were more frequently located in tightly packed regions of the protein structure (OSP>0.4) and solvent-inaccessible regions (RSA < 20%), suggesting that they may disrupt proteins’ core structural stability ^39^. In contrast, susceptible mutations were more often in less tightly packed, solvent-accessible regions. Additionally, resistance mutations exhibited higher conservation grades (5–9) than susceptible and background mutations, indicating their critical role in maintaining essential protein functions. These findings align with the hypothesis that resistance mutations often occur at functionally or structurally critical residues under strong evolutionary constraints ^40^. Understanding these distinctions could inform drug design by prioritising highly conserved and structurally significant target sites.

The stability analysis demonstrated variations in the predictive capabilities of different computational tools. While SDM and FoldX did not identify significant differences in ΔΔG values between resistant and susceptible mutations, PyRosetta revealed substantial differences. Resistant mutations showed a relatively more destabilising effect (median ΔΔG= −52.74) compared to susceptible mutations (median ΔΔG= −62.28), suggesting that PyRosetta may be more sensitive. These destabilising effects may reflect structural alterations that disrupt drug-binding interactions or enable conformational changes, thereby reducing drug efficacy.

Grouping mutations by first-line anti-TB drugs and MTBC lineages provided further resolution. For example, PyRosetta detected significant ΔΔG differences for NIH mutations in lineage 6 and EMB for Lineage 4, while FoldX highlighted significant differences for RIF (Lineage 6) and EMB and PZA (Lineage 2). These lineage-specific findings underscore the importance of considering genetic background and selective pressures when studying resistance mechanisms and designing treatment strategies.

While this study offers valuable insights into the genomic landscape of MTBC isolates in The Gambia, several limitations must be acknowledged. First, there is a potential underestimation of lineage 6 prevalence, as genotyping was primarily conducted from subcultured isolates, which may introduce culture bias against the slow-growing L6 isolation. Future studies could consider directly genotyping from sputum samples, minimising the culture bias associated with subculturing from freezer-stored isolates. Second, the functional significance of many identified mutations, particularly those classified as having “uncertain significance,” requires further investigation to clarify their clinical relevance. Experimental validation through in vitro and in vivo studies is essential. Thirdly, while tools like AlphaFold provided valuable insights, their accuracy in predicting the effect of mutations on protein stability (ΔΔG) remains limited ^41^. Integrating multiple predictive methods with experimental validation will enhance our understanding of mutation impacts. Lastly, only some of the WGS isolates had phenotypic resistance data, which limits our ability to confirm these mutations’ importance in drug resistance pathways.

Despite these limitations, this study highlights the importance of lineage-specific mutations providing a robust framework for future research. Addressing these gaps will improve our understanding of the genomic and structural mechanisms underlying DR and inform more effective TB control strategies.

## Conclusion

Our genome-wide analysis of *Mycobacterium tuberculosis* complex (MTBC) strains in The Gambia reveals a diverse landscape of circulating lineages and drug resistance profiles. L4 and L6 dominate the region, with L4 exhibiting global adaptability and virulence, while L6 remains regionally confined to West Africa. The coexistence of global and local lineages underscores the need for tailored, region-specific TB control strategies.

Drug resistance mutations, such as katG Ser315Thr and embC Ala307Thr, were notably frequent. Lineage-specific variants like embC Ala307Thr in L6 warrant further investigation for their roles in ethambutol resistance and bacterial fitness. Structural analyses highlighted that resistance mutations often occur in solvent-inaccessible, highly conserved regions, impacting protein stability and evolutionary fitness. These findings underscore the complexity of resistance mechanisms and their implications for treatment outcomes.

Finally, our study emphasises the importance of integrating genomic surveillance with functional validation to bridge the gap between genomic data and clinical outcomes. By refining our understanding of MTBC evolution, epidemiology, and resistance mechanisms, these efforts will inform tailored strategies to combat TB in The Gambia and contribute to global efforts to end TB.

## Figures and Tables

**Table 1:** Characteristics of the MTBC strains and underlying patients (N = 1803)

Characteristics	Number of isolates (N)	Percentage (%)
Year of diagnosis		
2002–2011	38	2.11
2012	338	18.75
2013	471	26.12
2014	585	32.45
2015	39	2.16
2016	26	1.44
2017	23	1.28
2018	94	5.21
2019	93	5.16
2020	6	0.33
NA	90	4.99
Age groups(yrs)		
Under 18	18	0.9
18–29	696	38.6
30–44	551	30.5
45 and above	287	15.9
NA	251	13.9
Sex		
Female	440	24.4
Male	1145	63.5
NA	218	12
Lineage		
L4	1214	67.2
L6	480	26.6
L2	59	3.2
L1	25	1.3
L3	21	1.1
L5	4	0.2
Genotypic drug res.		
Pan sensitive	1421	78
RIF	10	0.5
INH	90	5
MDR	19	1
Other	282	15.5

RIF = Rifampicin, INH = Isoniazid, MDR = multi-drug resistance, Other = Known drug resistance mutation but classified as uncertain significant by the WHO catalogue

**Table 2:** Known resistance markers in the first-line drugs

Drug	Gene	Mutation	Frequencies in The Gambia (%)	West-African frequencies (%)	Global frequencies (%)	Lineage (L)
Rifampicin	rpoB	Thr350Ile	26.9	50.9	0.99	6
		c.-199C > T	1.79	NA	6.75	4
		Ser450Leu	0.44	28.4	34.5	2,3,4
		Asp435Val	0.44	7.43	3.01	4
		Ile770Val	1.6	NA	0.11	4,6
Isoniazid	katG	Ser315Thr	3.84	48.34	39.34	2,3,4,6
		c.-497delA	4.07	NA	0.02	
	inhA	c.-777C > T	0.72	6.32	13.33	
		c.-154G > A	0.33	9.23	1.86	4,6
	ahpC	Pro44Arg	1.79	11.7	12.17	4
Ethambutol	embA	Thr113Arg	6.02	50.74	0.45	6
	embC	Ala307Thr	15.73	NA	0.05	6
	embB	Met306Val	0.19	3.66	13.85	2,4
		Met306Ile	0.14	7.02	12.32	4
Pyrazinamide	clpC1	Pro766Leu	1.65	2.74	7.60	4
	pncA	Leu172Pro	0.19	2.22	0.61	
		Asp63Ala	0.14	NA	0.97	
		His57Asp	0.04	NA	10	

## Data Availability

All the data used in the analysis presented will be uploaded into a dedicated space, the link of which will be published online together with the manuscript for open access to other researchers.
